# Expression profiles revealed potential kidney injury caused by SARS-CoV-2: a systematic analysis of ACE2 and clinical lessons learned from this discovery

**DOI:** 10.18632/aging.202224

**Published:** 2020-11-21

**Authors:** Jukun Song, Jianguo Zhu, Weiming Chen, Guohua Zhu, Wei Wang, Chi Chen, Zhenyu Jia, Yan Zha, Ping Xu, Zheng Wang, Fa Sun, Xiangyan Zhang

**Affiliations:** 1 Department of Oral and Maxillofacial Surgery, Guizhou Provincial People’s Hospital, Guizhou, China; 2 Department of Urology, Guizhou Provincial People’s Hospital, Guizhou, China; 3 Department of Immunology and Microbiology, Guiyang College of Traditional Chinese Medicine, Guiyang, Guizhou, China; 4 Department of Botany and Plant Sciences, University of California, Riverside, CA 92507, USA; 5 Department of Nephrology, Guizhou Provincial People's Hospital, Guizhou, China; 6 The Second Hospital of Wuhan Iron and Steel Group Corporation, Hubei, China; 7 Department of Respiratory and Critical Care Medicine, Guizhou Provincial People's Hospital, Guizhou, China

**Keywords:** COVID-19, ACE2, kidney injury

## Abstract

Background: Novel Coronavirus disease 2019 (COVID-19) was first detected in pneumonia patients in Wuhan, China in December 2019. Based on the current understanding, COVID-19 has become a global issue. Presumably, numerous studies have found that SARS-CoV-2 also transpires in kidney tissue with permanent viral loads. However, it is elusive as to whether SARS-CoV-2 can directly damage the kidney or induce acute renal failure. Hence, to comprehensively understand the impact of COVID-19 on kidney damage, we conducted a retrospective series of case studies to assess kidney functions. Additionally, ACE2 distribution in kidney tissue was analyzed through RNAseq data in open-access databases.

Results: According to the findings from transcriptome analysis, we revealed higher ACE2 expression levels in females than males. Similar results were more noticeable in the elderly than in young adults. Furthermore, single-cell RNA sequencing data analysis showed high ACE2 expression in kidney tubule and collecting duct principal cells as well as glomerular parietal epithelial cells. On their admission, the patient's serum creatinine and blood urea nitrogen (BUN) were elevated to between 36.13% and 16.80%, respectively. The estimated glomerular filtration rate (EGFR) of < 60 ml/min per 1.73 m2 was reported in 10.92 % of the patients. Notably, at admission, increased BUN time varied linearly following the generalized additive mixed model. Thus, the hourly-increase of BUN in patients was 0.495 (95%CI: 0.263, 0.726).

Conclusion: Based on clinical findings, it was ascertained that COVID-19 can damage renal function, but it seldom causes acute renal failure. Coronavirus may directly bind to ACE2-positive cells and damage kidney tissue in the analysis of scRNA-seq data in kidney tissue. Therefore, this evidence suggests that kidney tissue act as the SARS-CoV-2 infection site and the findings could provide insight into the pathophysiology of kidney damage.

Methods: We systematically analyzed ACE2 expression profiles in organs based on open-access datasets for healthy individuals. Meanwhile, single-cell sequencing data for kidney samples were collected and analyzed. Assessments on kidney functions were conducted on 119 selected COVID-19 positive patients admitted from 10^th^ February – 18^th^ March 2020, in hospital in Wuhan City, Hubei Province. Consequently, their clinical records and laboratory findings, such as the estimated glomerular filtration rate (eGFR), Blood Urea Nitrogen (BUN), Creatinine, and Comorbidities, were collected.

## INTRODUCTION

In early December 2019, several unknown pneumonia cases were reported in Wuhan City, Hubei Province, China [1, 2]. Following the sequencing on the patient's respiratory tract samples, the results revealed the presence of Coronavirus disease 2019 (COVID-19). As a result, the COVID-19 outbreak caused significant mortality and morbidity in China. As of February 11, 2020, China reported 72,314 individuals positive for COVID-19, a death toll of 1,023 was confirmed and over 21,675 suspected cases. Furthermore, globally, there were rapidly-growing numbers of COVID-19 patients [3]. The World Health Organization designated a global emergency health event as a result of major illnesses and deaths caused by the COVID-19 epidemic [4]. From December 2019, onwards, the spread of COVID-19 has become implacable thus attained epidemiological criteria to be declared a pandemic, having infected over 170,000 people in 100 countries and regions. COVID-19 is currently considered a global issue [5, 6].

Studies have demonstrated that a notable metallopeptidase called angiotensin-converting enzyme 2 (ACE2), is a functional receptor for Severe Acute Respiratory Syndrome coronavirus (SARS-CoV) [7]. Despite the natural role of SARS-CoV in the renin-angiotensin system, it gains entry into the cells through ACE2 [8, 9]. Besides, recent studies have shown that new coronavirus belongs to the Coronavirus family β-CoV, which is closely related to SARS-CoV [10, 11]. Meanwhile, the genome of severe acute respiratory syndrome coronavirus 2(SARS-CoV-2) has a similar sequence to SARS-CoV [12, 13]. As a result, it is hypothesized that new coronavirus and SARS-CoV enters the cell via the same ACE2 receptor. Notably, from recent assessments, it has been confirmed that ACE2 could be the host receptor for SARS-CoV-2 [14, 15]. However, potential avenues for SARS-CoV-2 infections as confirmed by bioinformatics evidence, are respiratory, cardiovascular, digestive, and urinary systems [16–18]. The expression of ACE2 in human kidney epithelial cells has also been extensively explored [19, 20]. It is therefore evident from these studies that, COVID-19 patients may cause kidney injuries. Nonetheless, the specific mechanisms underlying kidney damages caused by SARS-CoV-2 remain elusive especially on the gender disparity aspect [21]. Identification of the possible mechanism of kidney injury and urine transmission pathway is essential to elucidate the pathogenesis of COVID-19, thus providing early intervention to prevent kidney damage and cross-infection in patients with COVID-19, especially in asymptomatic virus carriers. Herein, we conducted a retrospective case series study to evaluate the renal function among COVID-19 patients. Of note, bioinformatics results from the ACE2 distribution in the kidney tissue discovered the potential mechanism of kidney damages. Also, the disparities related to race, age, gender, and smoking status in ACE2 expression levels were explored.

## RESULTS

### Tissue ACE2 expression analysis in the healthy population

**Figure 1:**
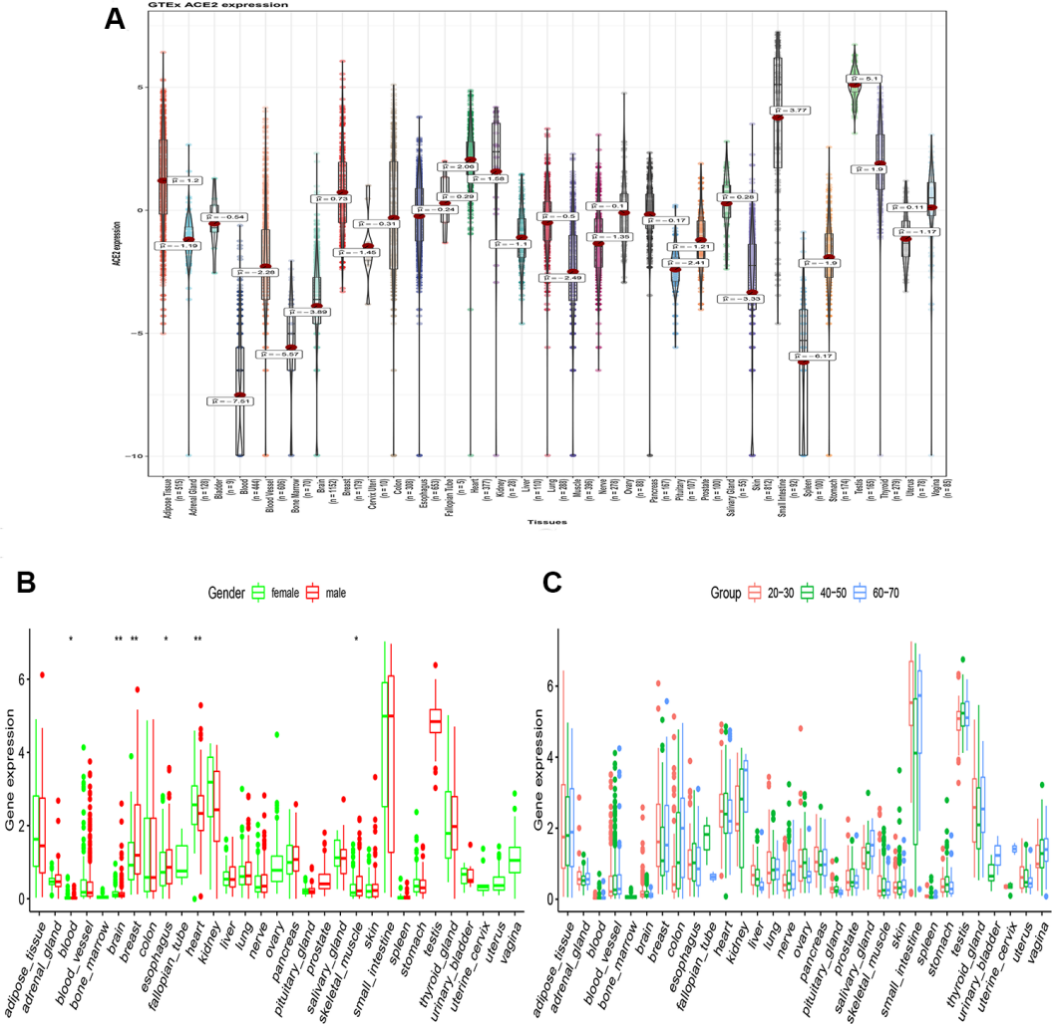
**RNA-seq analysis of public GTEx datasets.** (**A**) Violin plot of ACE2 expression in normal tissues, colored by organs, Kruskal–Wallis test was used to examine the difference across organs; (**B**) Box plot of ACE2 expression exhibits the gender disparity across different organs using Wilcox test; (**C**) Box plot of ACE2 expression exhibits the difference in different age populations using Kruskal–Wallis test. All P value less than 0.05 was considered statistically significant.

Among the COVID-19 patients, most have injuries on lung, kidney, testis, and oral mucosa. Therefore, urgency in exploring whether some organs are more vulnerable to COVID-19 in a healthy population is paramount. As a result, analysis of GTEx project data revealed that ACE2 was highly expressed in the kidney, digestive tract, lung, and testis (Figure 1A). However, the expression level of ACE2 was significantly altered between the male and female populations in the blood, brain, breast, heart, esophagus, and skeletal muscle organ. Notably, the ACE2 expression level was higher in the female population in the kidney, although it was not statistically significant (Figure 1B). Hence, the above result is consistent with the epidemiological characteristics of over 70,000 cases released by the Chinese Centers for Disease Control and Prevention on February 11, 2020 (The ratio is 0.99: 1 in Wuhan and 1.06: 1 for the whole country) [3]. According to our study, we found that the expression level of ACE2 in the kidney was positively correlated with age. Moreover, the ACE2 expression levels in the kidney were relatively higher in the elderly in most organs as compared to the children (Figure 1C). As a notable observation from this study, it may be partly explained that the elderly are highly susceptible to coronary pneumonia [2].

### Functional enrichment analysis

**Figure 2:**
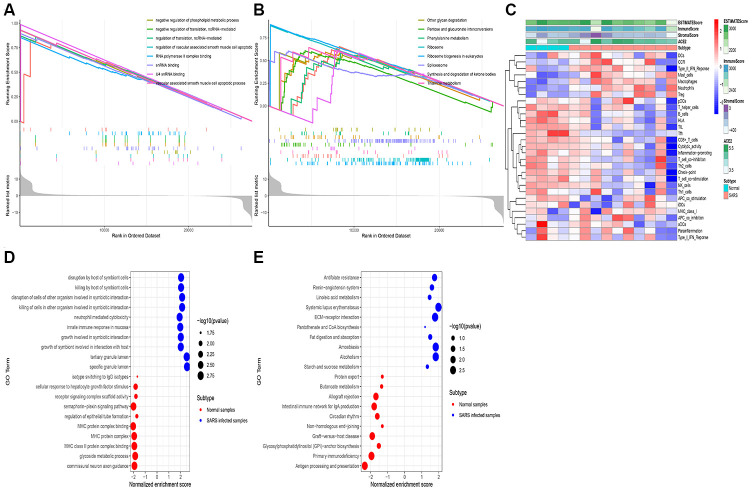
**RNA-seq analysis of the open-access dataset.** Functional enrichment analysis using the GSEA method in GTEx kidney samples exhibited the top 10 GO terms (**A**) and the top 10 KEGG terms (**B**). The immune landscape of severe acute respiratory syndrome (GSE 1739) (**C**), The GO terms (**D**), and The KEGG terms (**E**) were exhibited.

Gene expression profiles of 28 healthy populations from extracted from the GTEx dataset to examine the potential biological processes correlated with ACE2. Based on the median of ACE2 expression level, the samples were divided into two groups: higher expression and lower expression groups. Therefore, the GSEA was conducted to reveal the ACE2 related functional enrichment categories. Accordingly, our results revealed that enrichment of GO terms mostly occurred in the vascular associated smooth muscle cell apoptotic process, snRNA binding, and RNA polymerase II complex binding (Figure 2A). Furthermore, KEGG analysis demonstrated that ribosome biogenesis in eukaryotes, synthesis, and degradation of ketone bodies and spliceosomes were activated in the higher expression group (Figure 2B).

In the process of exploring whether the above activities can be triggered after coronavirus infection, we analyzed the expression profile of peripheral blood infected with SARS-CoV using the ssGSVA and GSEA method. Besides, the ssGSVA was employed to evaluate the immune cell infiltration in samples with infected SARS cells. Thus, the findings indicated that DCs (dendritic cells), mast cells, macrophages were elevated in the SARS infected cells. Meanwhile, both ACE2 expression in the tumor and the Immune Score increased simultaneously (Figure 2C). Additionally, the GSEA analysis revealed that several innate immune responses, host immune response was up-regulated in the samples with infected SARS cells, including disruption by a host of symbiont cells, neutrophil-mediated cytotoxicity, and tertiary granule lumen (Figure 2D, 2E).

These results also reveal that the upregulation of ACE2 can mediate the inflammatory response induced by cytokines. Concurrently, we speculated that the high ACE2 expression could prolong the virus life cycle, enhanced its replication ability, and mediate the invasion of coronavirus into host cells.

### Cancer cell line ACE2 expression analysis

**Figure 3:**
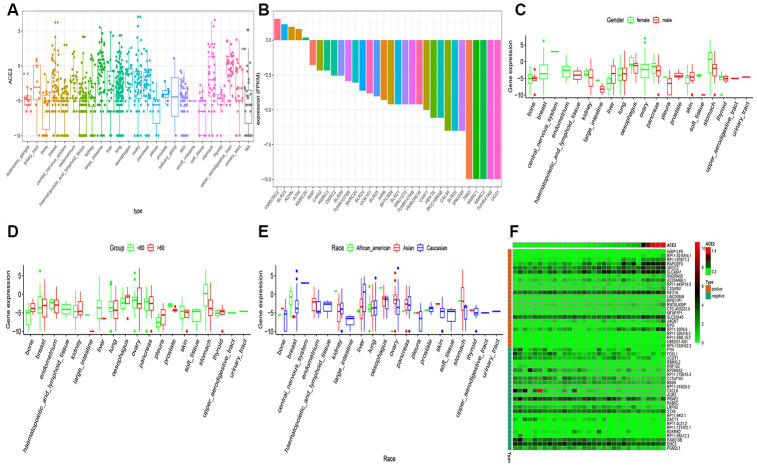
**RNA-seq analysis of public CCLE datasets.** (**A**) The Box plot exhibits of ACE2 expression in childhood tumor; (**B**) The Box plot exhibits of ACE2 expression in kidney cancer cell line; The disparities of gender (**C**), age (**D**) and race (**E**) was shown in the CCLE dataset. (**F**) The heatmap exhibits the top 25 positively/negatively related to ACE2 expression. Statistical significance was detected using Wilcox test for comparisons between two groups and Kruskal–Wallis test for more than two groups.

The gene expression profiles of the cancer cell line were downloaded from the CCLE dataset. The ACE2 expression levels were higher in the kidney, nasal mucosa, respiratory tract, bronchus, and lung (Figure 3A). Among the kidney cancer cell line, VMRCRCZ and SLR23 cell line has higher ACE2 expression levels (Figure 3B). Compared to the male population, the ACE2 expression level was higher in the female population in the kidney. However, statistical significance was not observed (Figure 3C). These findings are consistent with the previous observations, where the gender disparity was not detected. Also, we revealed that the ACE2 expression levels in the kidney cancer cell lines were positively correlated with age. The expression level of ACE2 in the kidney in the elderly was higher than in children (Figure 3D). on the contrary, ACE2 expression levels of Asians was not significantly higher than other races (Figure 3E).

The expression correlation between ACE2 and mRNAs were examined by calculating the Pearson correlation coefficient through mRNA expression profiles in 31 kidney cell lines. As a result, 2,485 protein-coding genes were expressed as highly correlated with ACE2 (Pearson correlation coefficient >0.5 and p < 0.001). Figure 3F exhibited the top 50 co-expressed genes.

### Analysis of ACE2 expression level in the TCGA dataset

**Figure 4:**
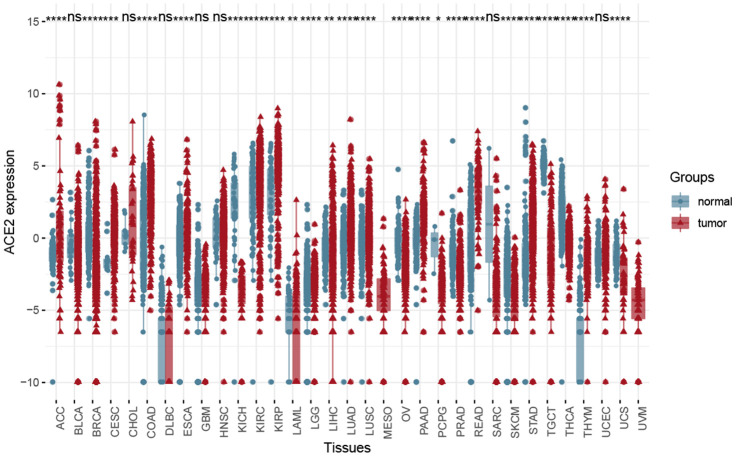
**The Box plot showed the difference between solid tumor-normal samples and tumor samples in the Pan-cancer level using Wilcox test.** (*P < 0.05, **P < 0.01; ***P < 0.001; ns, P >0.05).

The distribution of ACE2 expression levels in the pan-cancer in the TCGA dataset is shown in Figure 4. From the results, the differences between primary tumor and solid tissue normal samples were observed, such as KIRP and KIRP, KICH. Besides, the ACE2 expression in tumor samples was higher in solid normal samples in most tumors.

Accordingly, results have shown that KIRP and KIRC, KICH may be more closely related to the types of kidney cells infected by SARS-CoV and SARS-CoV-2. Meanwhile, ACE2 had a higher expression level in KIRP and KIRC samples, therefore our next study focused on tumor samples. Merging data of primary tumor samples and solid tissue normal samples by batch normalization using “sva” package increased the sample size and statistical power [27].

**Figure 5:**
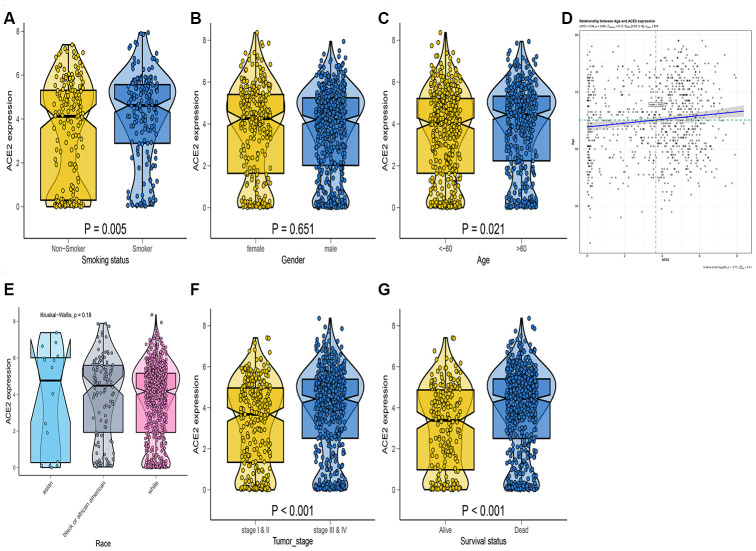
**RNA-seq analysis of public TCGA datasets.** Box-Violin plot shown the disparities of smoking status (**A**), gender (**B**), age (**C**), race (**E**), tumor stage (**F**), and survival probability (**G**). Two-tailed statistical P values were calculated by Wilcox test. (**D**) The association between age and ACE2 expression level.

In the tumor samples, ACE2 expression was up-regulated in smokers (Figure 5A), suggesting that smoking may increase the susceptibility of new coronaviruses. The ACE2 expression level was higher in the female population in the kidney. However, no statistical significance was observed (Figure 5B). We also found that the expression level of ACE2 was positively correlated with age. On the elderly, the expression level of ACE2 in the kidney was higher than in children (Figure 5C, 5D). Compared with other races, The analysis demonstrated that the ACE2 expression level in Asians was not significantly higher than of other races (Figure 5E). Thus, the expression level of ACE2 was associated with survival probability or pathological stage (Figure 5F, 5G). As a result, the expression of ACE2 in tumor patients is generally high and more susceptible to COVID-19 infection with a worse prognosis. Previous studies concur with our recent findings [28].

### Analysis of scRNA-seq data in kidney tissue

**Figure 6:**
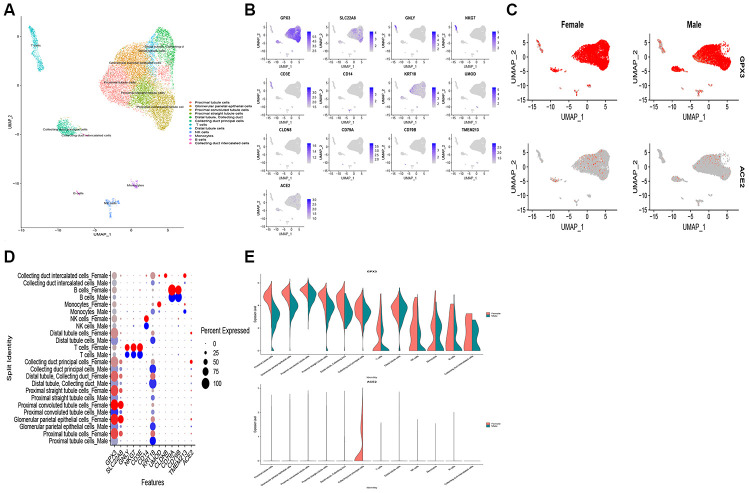
**Single-cell RNA-seq analysis of healthy kidney tissues in humans from publicly dataset.** (**A**) Twelve-cell types were identified by the cell markers, cells were clustered by the UMAP method; (**B**) Scatter plots of all the cells with ACE2 and other gene expressions; The scatter plot (**C**), dot plot (**D**) and violin plot (**E**) exhibits the disparities of ACE2 expression in gender.

To assess the expression pattern of ACE2 in the human kidney, we analyzed a published scRNA-seq dataset in the GEO dataset (GSE 131685). In the human dataset, 19,168 cells passed standard quality control and were retained for further analyses. We divided single cells into 12 sub-clusters based on the canonical markers and cell classification in the original literature. Accordingly, our results indicated that ACE2 expressions exhibit higher expression in the kidney tubule, collecting duct principal cells, and glomerular parietal epithelial cells (Figure 6A, 6B). Contrastingly, ACE2 expression was not observed in immune cells. Hence, these results indicate that kidney cells are the potential targets of COVID-19. Also, we found ACE2 most actively expressed in the female population (Figure 6C–6E), which is consistent with our findings from transcriptome analysis.

### COVID-19 damage renal functions in clinical data

**Figure 7:**
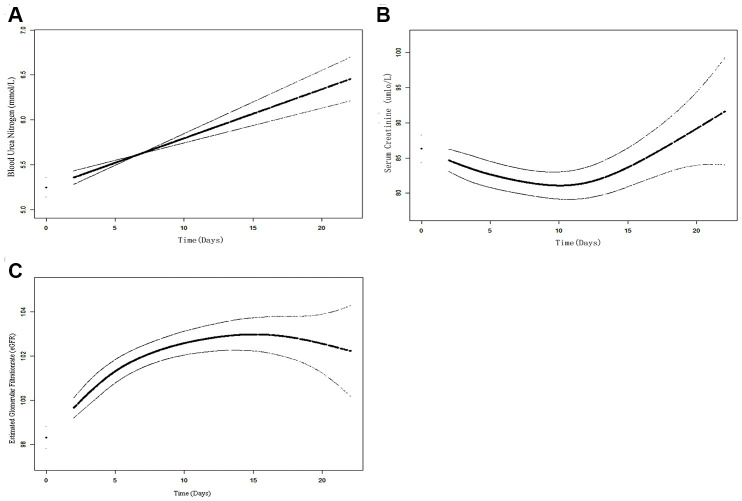
**The time-variation trend of kidney functions overtime during hospitalization using the GAMM model.** (**A**) The increased time-vary linear trend of BUN can be observed; (**B**) The increased time-vary non-linear trend of Cr; (**C**) The increased time-vary non-linear trend of eGRF.

Among 119 COVID-19 patients, there were 58 males and 61 females. The median age was 60 years and ranged from 20 to 84 years (Table 1). However, 8 critically-ill patients needed ventilator support to breathe in these cases. Only one patient was diagnosed with acute renal failure (ARF). Of the 119 COVID-19 patients, there were 3 death cases. After infection with SARS-CoV-2, serum creatinine, and blood urea nitrogen (BUN) were elevated in 36.13% and 16.80% of the patients on admission, respectively. Also, an estimated glomerular filtration rate < 60 ml/min per 1.73 m2 was reported in 10.92 % of patients. Notably, at admission, increased BUN time varies linearly following the generalized additive mixed model. Thus the hourly-increase of BUN in patients was 0.495 (95%CI: 0.263, 0.726) after adjusting for age, sex, comorbidities (diabetes and hypertension duration), and symptom (fever, cough) (Figure 7A). The non-linear association of eGFR/Creatinine was observed (Figure 7B, 7C). In general, as the length of inpatient treatment increases and so is the creatinine, even on patients who are about to be discharged. Therefore, these results suggest that COVID-19 patients continue to suffer kidney damage for a long time, and seldom develop ARF. Previous studies reported similar results [29].

**Table 1:** Characteristics and outcomes of patients with COVID-2019.

**Group**	**All patients**	**Normal baseline serum creatinine**	**Elevated baseline serum creatinine**	**P-value**
	**119**	**(N=76)**	**(N=43)**	****
**Age**				
≥60		46	19	0.044
<60		30	24	
**Gender**				
Male		38	23	0.035
Female		38	20	
**Coexisting Disorder**				
Hypertension		19	7	0.324
Diabetes		8	2	0.562
Cancer		1	1	0.256
COPD		3	1	0.458
Coronary heart disease		4	8	0.030

## DISCUSSION

Coronaviruses have caused two large-scale pandemics in the past two decades; SARS in 2002 and the Middle East respiratory syndrome (MERS) in 2012 [30]. Notably, in December 2019, COVID-19 broke out in Wuhan and rapidly spread to multiple regions and countries around the world. Precisely, it poses a major threat to global public health. With an increasing number of cases and expansion of the scope of infection, people were greatly concerned about the development of the epidemic [3]. Currently, COVID-19 patients were reported in over 100 countries and regions within weeks, thus becoming a public health emergency of global concern [5].

Similar to SARS-CoV infection, the spike (S) protein of SARS-CoV-2 was found to engage ACE2 as the host cellular receptors to enter host cells for reproduction and spread. To date, no effective treatment or vaccine has been clinically approved for these pathogens. The incubation period of COVID-19 is two weeks or longer, and it is highly contagious [7]. However, the main target organ is lung tissue, kidney tissue, testis tissue, and skin tissue. Although many studies have shown concerns with lung damage, estimates of kidney damage are often ignored. Besides, with the reinfection of COVID-19 after treatment, it has indicated that SARS-CoV-2 may exist in human organs for a long time. Therefore, we retrospectively analyzed laboratory kidney functions from 119 cases of COVID-19 patients and focusing on eGFR, BUN, and Creatinine. Clinical data results demonstrated that COVID-19 patients suffered from varying degrees of renal impairment. Therefore, to explore the specific mechanism of kidney damage in patients with COVID-19, we analyzed the expression pattern of ACE2 in kidney tissue using transcriptome data, and the findings revealed that kidney tissue may be the target for SARS-CoV-2. Herein, we investigated differences in ACE2 gene expression related to race, age, gender, and smoking status by analyzing transcriptome data in current work. According to our results, we found that ACE2 gene expression was significantly higher in the kidney tissue of former smokers than in nonsmokers. In the TARGET dataset, ACE2 expression was negatively correlated with age. In the elderly population, the opposite trend was observed. Therefore, more attention should be concerned with infants and elderly populations. From the TCGA and CCLE datasets, ACE2 expression was upregulated in smokers, suggesting that smoking may increase the susceptibility to COVID-19 infection. Although the higher expression of ACE2 was observed in the female population, no statistical significance was detected. These findings were consistent with the previous observation where gender disparity was not detected. We also found a higher expression level of ACE2 in the kidney of the elderly than on children. Therefore, further studies were needed to confirm these findings. Moreover, ACE2 was detected in all the samples, including a healthy population and adjacent tumor samples.

Recent studies have shown that kidney epithelial cells were found to be more infectious COVID-19 progeny than bronchial epithelial cells, therefore suggesting a viral kidney tropism [31]. Based on our current study of scRNA-seq data in adult kidney, we believe that kidney tissue may also be vulnerable to COVID-19 infection. The findings demonstrated that ACE2 was mainly enriched in renal cells, particularly in tubular cells, which was similar to a previous study [9]. According to Hamming et. al, they reported that ACE2 was present in the glomerular visceral and parietal epithelium, brush border and cytoplasm of proximal tubular cells, and the cytoplasm of distal tubules and collecting ducts [9]. Renal tubular cells have reabsorption functions, excretion of metabolites, maintaining fluid balance and acid-base balance. Meanwhile, several studieshave successfully tested virus RNA from the urine of COVID-19 patients, and suggested the possibility reservoirs of kidney tissue for SARS-CoV-2 [29].

Currently, researchers are keen on COVID-19 patient’s lung symptoms while neglecting other symptoms like kidney damage, gastrointestinal symptoms. For example in Mycobacterium tuberculosis (TB), COVID-19 can not only cause respiratory infections, but also damage other organs, such as the small intestine, kidney, and brain. Therefore, we should treat COVID-19 patients like TB patients. Cheng’s research team has reported a high rate of renal abnormalities in COVID-19-positive patients. The prevalence of elevated serum creatinine, elevated blood urea nitrogen and estimated glomerular filtration under 60 ml/min/1.73m2 were 14.4, 13.1, and 13.1%, respectively [32]. The clinical data demonstrated that SARS-CoV-2 directly infects human renal tubules and may cause kidney damage. Furthermore, a recent study has reported that detection of viral RNA in urine samples from patients with severe SARS-CoV-2 infection [33], suggesting that kidney-derived virus particles may pass through the kidney glomerulus to enter the urine. Similarly, the results agree with our study that ACE2 can bind to glomerular parietal epithelial cells. Therefore, SARS-CoV-2 can enter renal tubular cells by binding to ACE2, thereby inducing cytotoxicity and renal dysfunction.

In this recent study, clinical data demonstrated that COVID-19 patients suffered from different degrees of kidney damage, even ARF. Meanwhile, we used the transcriptome data to analyze the distribution of ACE2 expression in races, ages, and genders in the kidney. The results of scRNA-seq data have accurately located the expression and distribution of ACE2 in kidney cells. Therefore, these findings provide evidence that human kidneys are potential targets for SARS-CoV-2, which could provide a piece of proof for the future prevention strategy of kidney damage in clinical practice as well as daily life. However, the specific mechanism of its potential kidney damage is still unclear and should be further explored.

## MATERIALS AND METHODS

### Transcriptome data

Profiles of gene expression were downloaded from the TCGA kidney cohort (https://portal.gdc.cancer.gov/), Genotype-Tissue Expression (GTEx) project (https://commonfund.nih.gov/GTEx/), Gene Expression Omnibus (GEO) (https://www.ncbi.nlm.nih.gov/geo/) and Broad Institute Cancer Cell Line Encyclopedia (CCLE) (https://portals.broadinstitute.org/ccle/) open-access datasets. The mRNA data on normal kidney was obtained from the GTEX project, which included 28 normal kidney tissues. Besides, the ACE2 expression distribution across different organs was explored. Assessment of TCGA three kidney cohorts (KICH, KIRC, and KIRP) was enrolled to unveil the disparities between normal healthy tissues and tumor samples. Thereafter, we merged the mRNA data in TCGA three kidney cohorts to one gene expression profile using the batch normalization. Consequently, the RNA-seq profiles (FPKM values) and phenotype data were downloaded. According to previous studies, the gene expression profile of severe acute respiratory syndrome (GSE 1739) data was collected from the GEO dataset which including 4 PBMC normal samples and SARS patient blood samples [22]. We also downloaded the Transcript data in the cancer cell line were downloaded from the CCLE dataset. Moreover, disparities related to race, age, gender, and smoking status in ACE2 gene expression was examined.

### Publicly available scRNA-seq datasets

The scRNA data of normal human kidney tissue was obtained from the GEO (GSE 131685), which included two males and a female population [23]. Exploration of gender difference in ACE2 expression was assessed through individual analysis on the scRNA dataset of male and female populations. Cell types were distinguished using Securat 3.1.4. After normalizing the data by the LogNormalize method, the first 2000 most highly variable feature genes (HVGs) were used in cell clustering analysis. Consequently, the cell types with high ACE2 expression levels were identified based on the scRNA-seq datasets. The cell scatter plot was used by UMAP method.

### Functional enrichment analysis

Herein, the determination of the overall pathway of gene-set activity score for each sample in the GTEx datasets was conducted through Gene Set Enrichment Analysis (GSEA) [24] and Gene Set Variation Analysis (GSVA) [25] of the normal kidney. Besides, gene sets using the c2/c5 curated signatures were downloaded from the Molecular Signature Database (MSigDB) of Broad Institute. However, the GO/KEGG terms were identified between highly ACE2 expressed and lowly ACE2 expressed groups. Therefore, a significant enrichment pathway was determined based on FDR < 0.05 where the common pathways in both datasets were chosen.

### Clinical data and data analysis

Through a retrospective case series study, 119 COVID-19 patients admitted to the Second Hospital of WUHAN iron and steel group corporation, Hubei Province from 10^th^ February - 18^th^ March 2020 were enrolled. Basic clinical information, such as age, gender, and comorbidities was collected from the enrolled COVID-19 patients. Moreover, on admission, the patients' laboratory test data such as estimated glomerular filtration rate (eGFR), Blood Urea Nitrogen (BUN) and Creatinine (Cr) were recorded.

The time-variation trend of kidney functions was analyzed using the Generalized additive mixed model (GAMM) on COVID-19 admitted patients [26]. However, the study protocol was approved by the Ethics Committee of the Second Hospital of Wuhan Iron and Steel group corporation, and the included patients should ascent for their consent in advance.

### Statistical analysis

Statistical significance was detected using Wilcox test for comparisons between two groups and Kruskal–Wallis test for more than two groups [18]. The correlation between ACE2 and target gene was analyzed using Pearson correlation analysis. The functional enrichment analysis was conducted by GSEA and GSVA. Generalized additive mixed model (GAMM) was used to evaluate the time-variation trend of kidney functions among COVID-19 admitted patients. All analysis was performed using R software (R-project.org), R packages obtained through the Bioconductor project (http://www.bioconductor.org/) were applied for statistical analysis. All P values were set as bilateral, and a P value <0.05 was regarded as statistically significant.

### Ethics approval

The ethical approval was authorized by the Wuhan Wugang Hospital.
